# Diagnostic model for hepatocellular carcinoma using small extracellular vesicle-propagated miRNA signatures

**DOI:** 10.3389/fmolb.2024.1419093

**Published:** 2024-06-28

**Authors:** Xinyi Luo, Lin Jiao, Qin Guo, Yi Chen, Nian Wang, Yang Wen, JiaJia Song, Hao Chen, Juan Zhou, Xingbo Song

**Affiliations:** ^1^ Department of Laboratory Medicine, West China Hospital, Sichuan University, Chengdu, China; ^2^ Department of Laboratory Medicine, the First People’s Hospital of Ziyang, Ziyang, China

**Keywords:** small extracellular vesicle, microRNA, biomarker, hepatocellular carcinoma, cancer

## Abstract

**Background:**

Hepatocellular carcinoma (HCC) is the most common type of liver cancer. Small extracellular vesicles (sEVs) are bilayer lipid membrane vesicles containing RNA that exhibit promising diagnostic and prognostic potential as cancer biomarkers.

**Aims:**

To establish a miRNA panel from peripheral blood for use as a noninvasive biomarker for the diagnosis of HCC.

**Methods:**

sEVs obtained from plasma were profiled using high-throughput sequencing. The identified differential miRNA expression patterns were subsequently validated using quantitative real-time polymerase chain reaction analysis.

**Results:**

The random forest method identified ten distinct miRNAs distinguishing HCC plasma from non-HCC plasma. During validation, miR-140-3p (*p* = 0.0001) and miR-3200-3p (*p* = 0.0017) exhibited significant downregulation. Enrichment analysis uncovered a notable correlation between the target genes of these miRNAs and cancer development. Utilizing logistic regression, we developed a diagnostic model incorporating these validated miRNAs. Receiver operating characteristic (ROC) curve analysis revealed an area under the curve (AUC) of 0.951, with a sensitivity of 90.1% and specificity of 87.8%.

**Conclusion:**

These aberrantly expressed miRNAs delivered by sEVs potentially contribute to HCC pathology and may serve as diagnostic biomarkers for HCC.

## 1 Introduction

HCC is a predominant form of primary liver cancer, accounting for 80% of cases globally (Llovet et al., 2021). Liver cancer continues to pose a significant threat to global public health. In 2022, liver cancer caused more than 757,000 deaths globally, making it the third most lethal cancer. Additionally, it is the sixth most commonly diagnosed cancer worldwide, with approximately 865,000 new cases reported during the same year (Bray et al., 2024). The incidence and mortality figures reflect the bleak outlook for the disease. Chronic HBV infection comprises approximately 50% of global cases, primarily in Sub-Saharan Africa and Asia (McGlynn et al., 2015). In addition to HBV, chronic hepatitis B (CHB) patients with elevated HCC risk are older; are male; have cirrhosis, diabetes mellitus, or environmental exposures; have excessive alcohol and tobacco use; and have coinciding HCV or HIV infection (McGlynn et al., 2015; Hung et al., 2017; Ozakyol, 2017; Xie, 2017; Petruzziello, 2018). Unfortunately, HCC is commonly diagnosed at an advanced stage because there are no detectable symptoms in the early stages of the disease, and there are no early diagnostic markers for the disease. It is imperative to develop reliable and precise molecular diagnostic biomarkers to facilitate early detection of HCC in light of these challenges.

Numerous types of cells secrete sEVs, which can be detected in almost all biological fluids as promising cancer biomarkers (van Niel et al., 2018). These sEVs carry genetic and molecular information, including proteins and nucleic acids such as microRNAs (miRNAs), messenger RNAs, and DNA, providing valuable insights into their cellular origin (Zhang and Yu, 2019). Compared to normal cells derived from the same tissue, cancer cells release a significantly greater quantity of sEVs (Sun et al., 2018). These vesicles are crucial for intercellular communication. miRNAs are small (∼22 nt long), noncoding RNAs that operate by modulating gene expression (Diener et al., 2022). Compared with those of parental cells, sEV-delivered miRNAs can exhibit distinct profiles (Yu et al., 2016). Abnormal expression of miR-21 and miR-221 in sEVs has been reported in the progression of CHB to HCC (Tomimaru et al., 2012; Wang et al., 2014; Li et al., 2015). Furthermore, research has revealed a significant suppression of sEV-delivered miR-718 after liver transplantation in patients with HCC recurrence (Sugimachi et al., 2015). It is possible to use sEV-delivered miRNAs as noninvasive biomarkers for early-stage HCC detection.

In our study, we noted significant differences in plasma sEV-delivered miRNA expression between individuals with HCC and those without HCC. Through verification of the validation cohort, we identified two feature miRNAs that exhibited the most informative characteristics. Based on our findings, we devised a novel model to differentiate HCC patients from healthy donors. This article showcases the initial results and will undergo additional validation with larger sample sizes before clinical implementation. We hope that our findings will aid in the development of promising HCC detection biomarkers.

## 2 Materials and methods

### 2.1 Patients and samples

Participants (study group, n = 20; validation group, n = 177) were recruited from West China Hospital, Sichuan University. In the study group, 15 patients—8 with CHB and 7 with HCC—were enrolled, along with 5 healthy controls. For the validation stage, an additional 126 patients—49 with CHB and 77 with HCC—were included, along with 51 healthy controls. Peripheral blood samples were collected from all patients prior to treatment, and the diagnosis of HCC or CHB was confirmed through pathological tissue analysis.

The fasted participants provided 10 mL of peripheral blood collected in EDTA-K2-coated tubes to prevent hemolysis or blood clotting. The blood was promptly centrifuged at 1,000 rpm for 5 min at 4°C to separate the plasma within 2 h, and the isolated plasma was stored at −80°C to prevent repeated freeze-thaw cycles. Patients who were xenotransplanted or had blood-related diseases, alcoholism, or other hepatitis virus infections were excluded. Patients in the control group underwent examinations during the same period and had no history of malignancy or hepatobiliary disease. All the samples were obtained with informed consent from the patients, and the study was approved by Biomedical Ethics Committee of West China Hospital of Sichuan University [Reference No. 2019 (203)].

### 2.2 Extraction of sEVs from peripheral blood

Two techniques were employed to extract sEVs according to the MISEV 2018 criteria (Théry et al., 2018), which aim to circumvent isolation-based biases. In the study group (six males and two females), sEVs were isolated using differential ultracentrifugation. The plasma was diluted 1:1 with PBS (Sangon, China) and filtered through a 0.22 μm syringe filter (Millipore, USA). The filtered solution was centrifuged at 15,000 × g for 30 min to eliminate other medium/large EVs and certain non-EV components (Optima XE, Type 70 Ti, k-factor: 368, Beckman Coulter, USA). The resulting supernatant was further centrifuged at 150,000 × g for 4 h (Optima MAX-XP, MLS-50, k-factor: 88, Beckman Coulter, USA). The pellet was washed with cold PBS and subjected to additional centrifugation at 150,000 × g for 2 h. The pellet was resuspended in PBS to create a suspension of sEVs. For the validation group, sEVs were extracted using the exoRNeasy Midi Kit (Qiagen, Germany). The specific steps were conducted according to the procedure outlined in the instruction manual. The sEV suspensions were immediately processed for downstream applications.

### 2.3 Transmission electron microscopy (TEM)

The sEV suspension was deposited onto a copper grid and allowed to settle for 1 min. Any surplus liquid was gently removed by wiping the grid with clean filter paper. The samples were then negatively stained with a 2% phosphotungstic acid solution before being allowed to air dry for several minutes. The grids were examined using a Philips CM100 Compustage (FEI) transmission electron microscope with an acceleration voltage of 80 kV, and digital images were captured.

### 2.4 Nanoparticle tracking analysis (NTA)

After being resuspended in PBS, the samples were diluted 1,000- to 5000-fold and manually injected into the sample chamber of the NTA instrument (NanoFCM, United Kingdom) at room temperature. The nanoparticles within the sample were illuminated by the laser, and their movements, driven by Brownian motion, were recorded and analyzed. This analysis enabled the measurement of the concentration and particle size distribution of sEVs.

### 2.5 Western blotting

The sEV suspensions were treated with RIPA buffer (Boster, USA). The proteins were separated and transferred to PVDF membranes. After a 1-h blocking step with 5% bovine serum albumin (BSA), the membranes were incubated overnight with the following primary antibodies: rabbit anti-CD63 monoclonal antibody (Abcam, United Kingdom; 1:1,000 dilution), rabbit anti-TSG-101 monoclonal antibody (Abcam, United Kingdom; 1:1,000 dilution), rabbit anti-APOB monoclonal antibody (R&D Systems, USA; 1:1,000 dilution), and rabbit anti-ASGPR1 monoclonal antibody (Santa Cruz, USA; 1:1,000 dilution). The membranes were then incubated with a 1:5,000 dilution of the corresponding secondary antibody (Pierce, USA) for 1 hour. Finally, an enhanced chemiluminescence kit (Beyotime, China) was used to observe the bands.

### 2.6 sEV-delivered RNA isolation, quality control and quantification

An exoRNeasy Midi Kit was used to isolate total RNA. The quality and quantity of the extracted sEV-delivered RNA were assessed using an Agilent 2,100 Bioanalyzer (Agilent Technologies, USA) and a Qubit Fluorometer (Thermo Fisher Scientific, USA).

### 2.7 Small RNA library construction and sequencing

The sEV-delivered RNA obtained previously underwent PAGE, followed by purification and recovery of the 18–30 nt fragments. Libraries were prepared by ligating 3′ and 5′ adaptors to the small RNA, followed by reverse transcription, PCR amplification, and size selection using polyacrylamide gels. An Agilent 2,100 Bioanalyzer (Agilent Technologies, USA) and an ABI StepOnePlus real-time PCR system (Applied Biosystems, USA) were used to quantify the library. Finally, the Illumina HiSeqTM 2,500 platform (Illumina, Inc., USA) was used to sequence the library.

### 2.8 Bioinformatics analysis

Clean reads obtained after adaptor and low-quality tag removal were utilized for bioinformatic analysis. To eliminate fragments of rRNA, tRNA, snRNA, and snoRNA, we screened against Rfam 11.0 and GenBank. The trimmed reads were aligned against miRBase V22 to identify existing miRNAs, while MIREAP was used to identify novel miRNAs with hairpin-like structures. Raw counts from the sequencing data were normalized to transcripts per million (TPM), and differentially expressed miRNAs (DEMs) across different groups were screened using the ‘edgeR’ package in R (Chen et al., 2016). The criteria for identifying DEMs were as follows: a log2-fold change greater than 1 or less than −1 and a *p*-value <0.05. Heatmaps and volcano maps illustrating the analysis results were generated using RStudio software (version 2023.06.0 + 421). miEAA was utilized to analyze the biological processes and pathways associated with the predicted DEMs.

The random forest algorithm is composed of multiple decision trees, each of which uses a random subset of parameters to create binary splits in the data for classification. These tree predictions are combined through majority voting to produce the final classification (Rigatti, 2017). The selection process was carried out using the random forest package within R.

### 2.9 Quantitative real-time RT‒PCR (qRT‒PCR)

Total RNA was extracted from the validation group, purified, and reverse transcribed to cDNA. The reverse transcription reaction mixture consisted of 2 μL of template RNA and 8 μL of 10x miRCURY RT enzyme mix (Qiagen, Germany). The reverse transcription process involved incubation at 42°C for 60 min, followed by heat inactivation of the reverse transcriptase at 95°C for 5 min and immediate cooling to 4°C. For qRT‒PCR, the miRCURY LNA SYBR Green PCR Kit (Qiagen, Germany) and the ABI 7500 qPCR system (Applied Biosystems, USA) were used with a reaction volume of 10 μL. The polymerase was activated at 95°C for 2 min, followed by 40 cycles of denaturation at 95°C for 10 s and primer annealing at 60°C for 60 s. Commercial probes, specifically miRCURY LNA miRNA PCR Assays (Qiagen, Germany), were utilized to detect the reference gene and target miRNA. The sequences of all primers used in this study are listed in Supplementary Table S3. Gene expression levels were calculated by the 2^−ΔΔCT^ method, with miR103a-3p serving as an internal control for miRNA. We performed qRT‒PCR on plasma samples from 51 healthy controls, 49 patients with CHB and 77 patients with HCC.

### 2.10 Functional annotations of the identified miRNAs

TargetScan, miRDB, and miRWalk were used to predict target genes. Next, these target genes were subjected to Gene Ontology (GO) enrichment analysis. The GO enrichment analysis provides valuable insights into the potential roles and functions of the miRNAs of interest.

### 2.11 Statistical analysis

IBM SPSS Statistics for Windows v25 (IBM Corp., USA) was used for data analysis, and GraphPad Prism 8.0.2 (GraphPad Software, Inc., USA) was used to create the experimental images. The association between DEMs and the risk of HCC was assessed using conditional logistic regression, and the Student’s t-test was utilized to compare the two groups via qRT‒PCR analysis. *p*-values less than 0.05 were deemed to signify statistical significance for differences. The AUC was used to evaluate DEMs’ prediction ability.

## 3 Results

### 3.1 Characterization of participants and sEVs isolated from the two groups

We recruited a study group comprising seven HCC patients, eight CHB patients, and five healthy donors. Additionally, a validation group with 77 HCC patients, 49 CHB patients, and 51 healthy donors was established. The clinicopathological characteristics of the patients enrolled in this study are shown in Table 1.

**TABLE 1:** Clinicopathological characteristics of HCC patients.

Clinicopathological characteristic		Study group n (%)	Validation group n (%)	*p*-value
Sex	Female	2 (29)	16 (21)	0.639
	Male	5 (71)	61 (79)	
Age (years)	≤60	3 (43)	50 (65)	0.415
	>60	4 (57)	27 (35)	
AFP (ng/mL)	≤20	4 (57)	30 (39)	0.433
	>20	3 (43)	47 (61)	
HBsAg	Negative	3 (43)	15 (20)	0.165
	Positive	4 (57)	62 (80)	
HCV	Negative	6 (86)	71 (92)	0.469
	Positive	1 (14)	6 (8)	
Liver cirrhosis	Absence	2 (29)	5 (6)	0.103
	Presence	5 (71)	72 (94)	
Tumor grade	G1	0 (0)	40 (52)	0.017
	G2	4 (57)	20 (26)	
	G3	3 (43)	17 (22)	

Abbreviations: AFP, alpha fetoprotein; HBsAg, hepatitis B surface antigen; HCV, hepatitis C virus.

**FIGURE 1:**
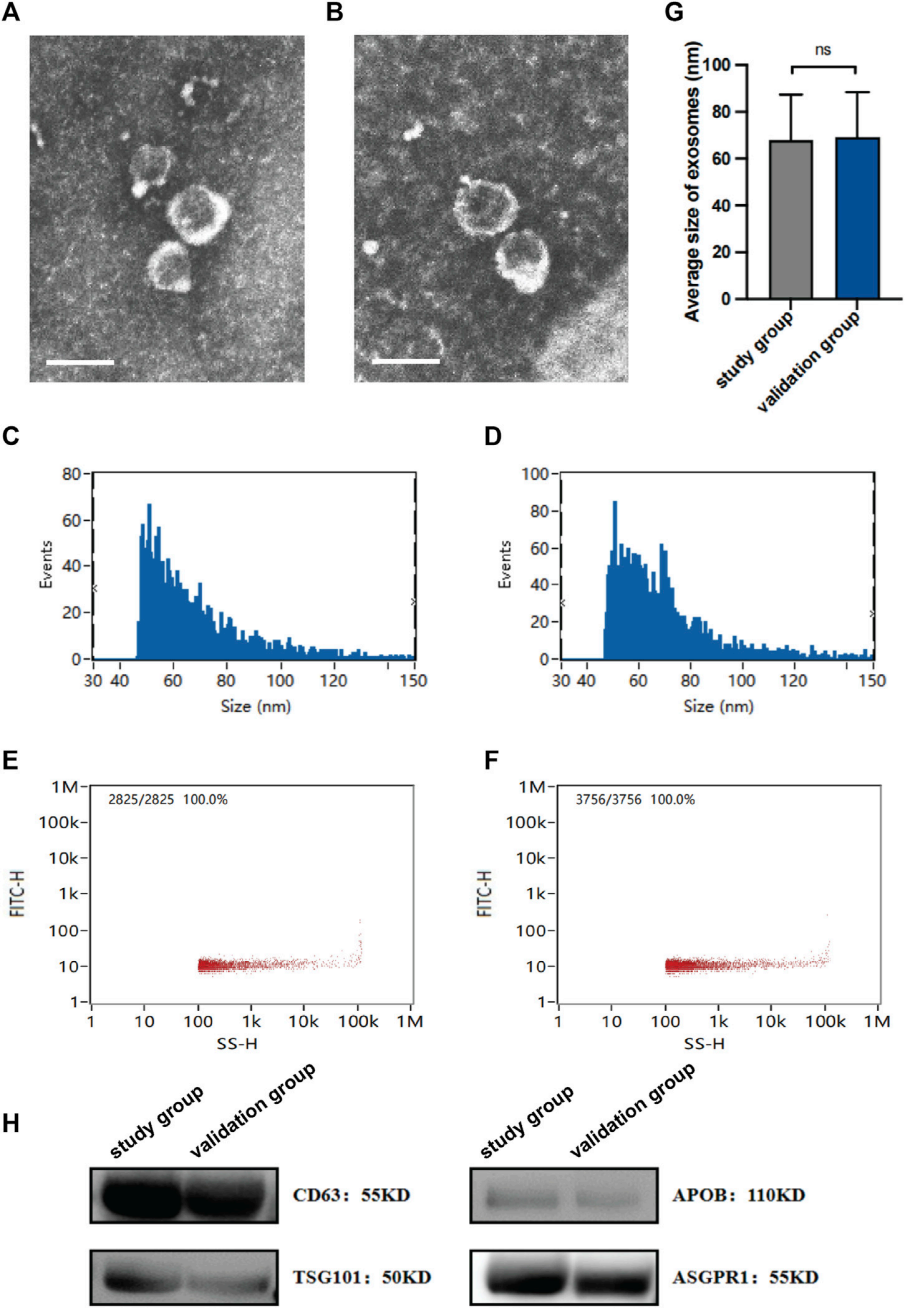
Characterization of isolated sEVs. The morphology of sEVs from the study group **(A)** and the validation group **(B)** was observed by TEM. Scales bars, 100 nm. The size of sEVs from the study group **(C)** and the validation group **(D)** was assessed by NTA. The concentration of sEVs from the study group **(E)** and the validation group **(F)** was assessed by NTA. **(G)** Average sizes of sEVs from the study group and the validation group. **(H)** Expression of protein markers of sEV, as evaluated by western blotting. Data are mean ± standard deviation (SD); ns: *p* > 0.05.

sEVs were isolated from the study group and the validation group using ultracentrifugation and the Qiagen exoRNeasy Midi Kit, respectively. TEM analysis of the plasma-derived sEVs revealed similar lipid bilayers and a typical cup shape (Figures 1A,B). Moreover, NTA demonstrated that both purified vesicle sets had an average diameter of approximately 70 nm (Figures 1C,D,G). The particle concentrations were measured at 1.35×10^10^ and 1.88×10^10^ particles/mL, respectively (Figures 1E,F). Furthermore, western blotting for sEV markers (CD63 and APOB), an endosomal marker (TSG101), and ASGPR1 (a hepatic marker gene) confirmed the successful isolation of sEVs from plasma, some of which originated from the liver (Figure 1H). Overall, the findings indicated consistency in the isolation and characterization of sEVs between the study and validation groups, with no significant differences observed.

### 3.2 Differentially expressed miRNAs and identification of feature miRNAs

**FIGURE 2:**
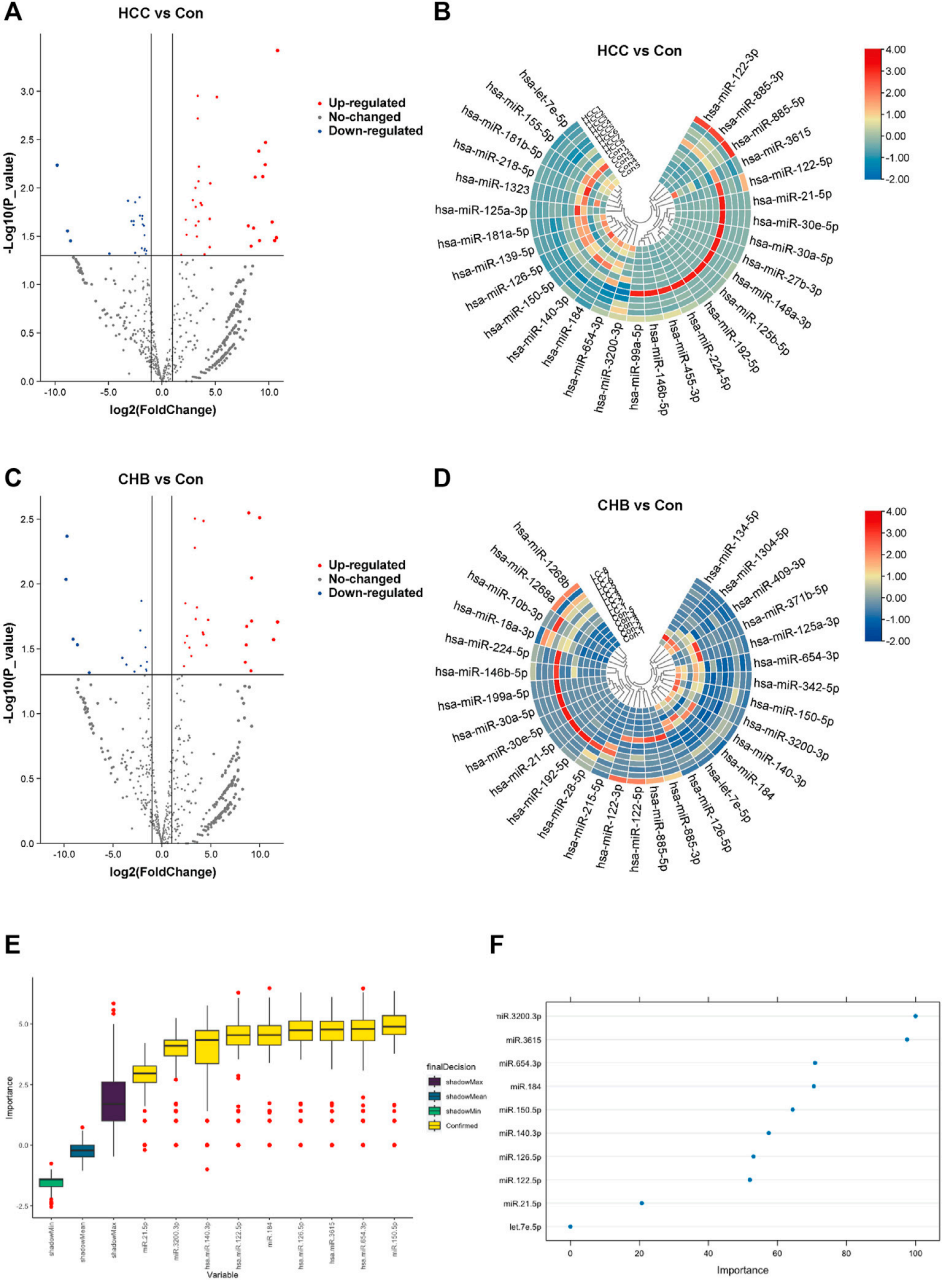
Aberrantly expressed miRNAs in HCC and CHB. **(A)** The volcano plot shows the expression of sEV-delivered miRNAs in HCC patients, with red and blue spots representing significantly upregulated and downregulated miRNAs in HCC, respectively. **(B)** Hierarchical clustering analysis of the sEV-delivered miRNA expression profile in HCC patients, displaying the top 30 enriched miRNAs. **(C)** The volcano plot represents the expression of sEV-delivered miRNAs in CHB patients. **(D)** Hierarchical clustering analysis of the sEV-delivered miRNA expression profile in CHB patients, showing the top 30 enriched miRNAs. **(E)** The boxplot displays the minimum, average, and maximum shadow scores of all combined features, with yellow variables representing important miRNAs confirmed by random forest analysis. **(F)** The dot plot visually represents the importance of variables. Each dot corresponds to a miRNA, and its position on the axis reflects its level of importance. Generally, more important miRNAs are positioned higher on the axis.

The miRNAs that were differentially expressed among these groups were examined. The most significant DEMs in sEVs from HCC patients are listed in Supplementary Table S1. Of the 55 DEMs, 22 were downregulated, and 33 were upregulated (Figures 2A,B). For the comparison between the CHB and healthy groups, the DEMs that exhibited significant alterations are presented in Supplementary Table S2. Among the 41 significantly altered miRNAs, 26 were upregulated and 15 were downregulated (Figures 2C,D).

In our analysis, we applied the random forest algorithm to identify feature miRNAs from the pool of DEMs. The results revealed that 9 miRNAs exhibited significant predictive power and were identified as important attributes: miR-21-5p, miR-122-5p, miR-126-5p, miR-140-3p, miR-150-5p, miR-184, miR-654-3p, miR-3615, and miR-3200-3p (Figure 2E). Additionally, we identified one miRNA, let-7e-5p, as a potential attribute that requires further investigation (Figure 2F).

### 3.3 Verification of the differentially expressed candidate miRNAs

**FIGURE 3:**
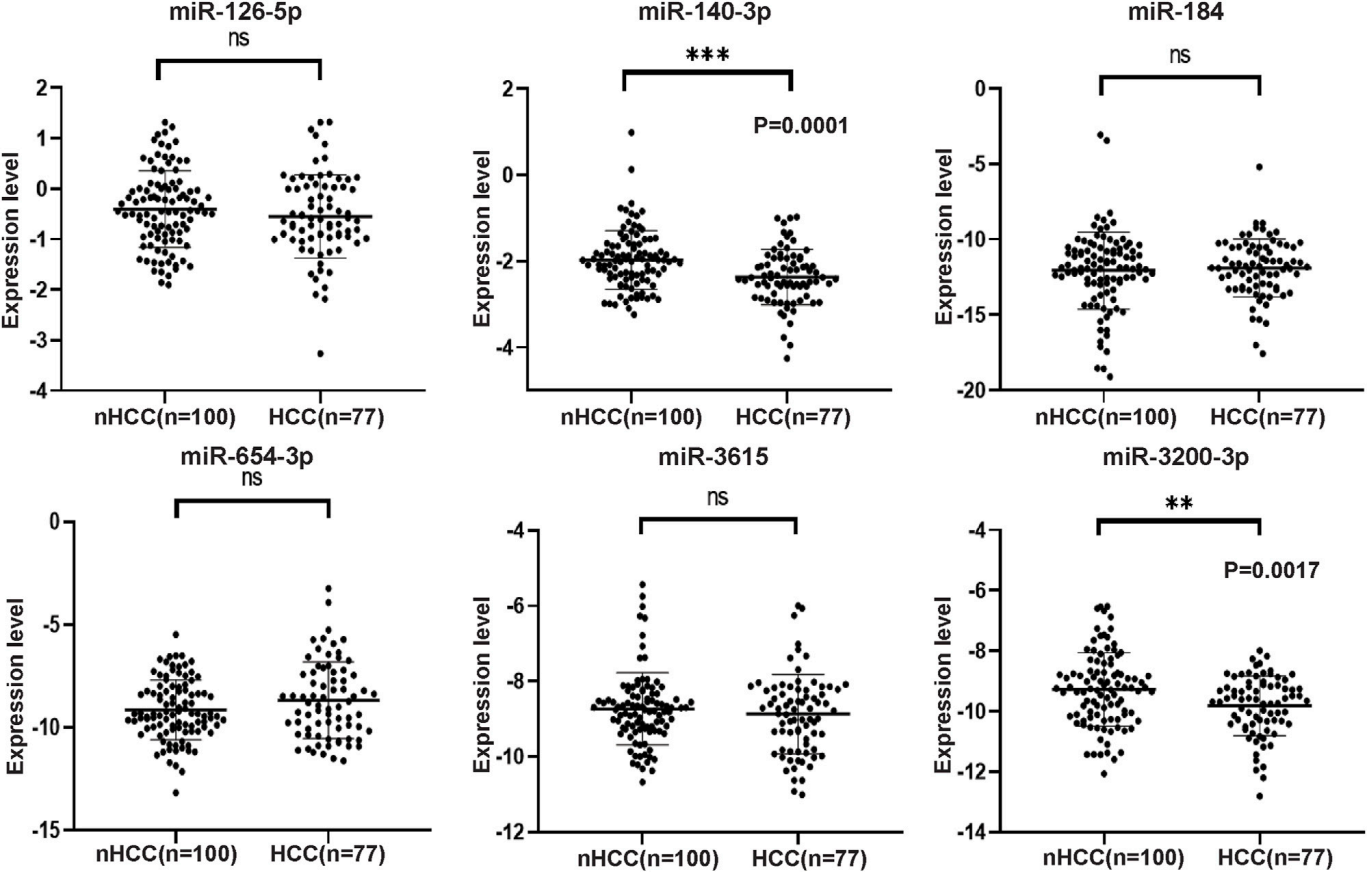
The expression levels of six selected miRNAs analyzed by qRT-PCR in HCC and nHCC samples. The expression values were normalized to miR103a-3p, and the experiments were performed three times. The results revealed that miR-140-3p and miR-3200-3p exhibited downregulation in the plasma sEVs of HCC patients. However, there were no significant changes observed in the expression levels of miR-126-5p, miR-184, miR-654-3p, and miR-3615.

**FIGURE 4:**
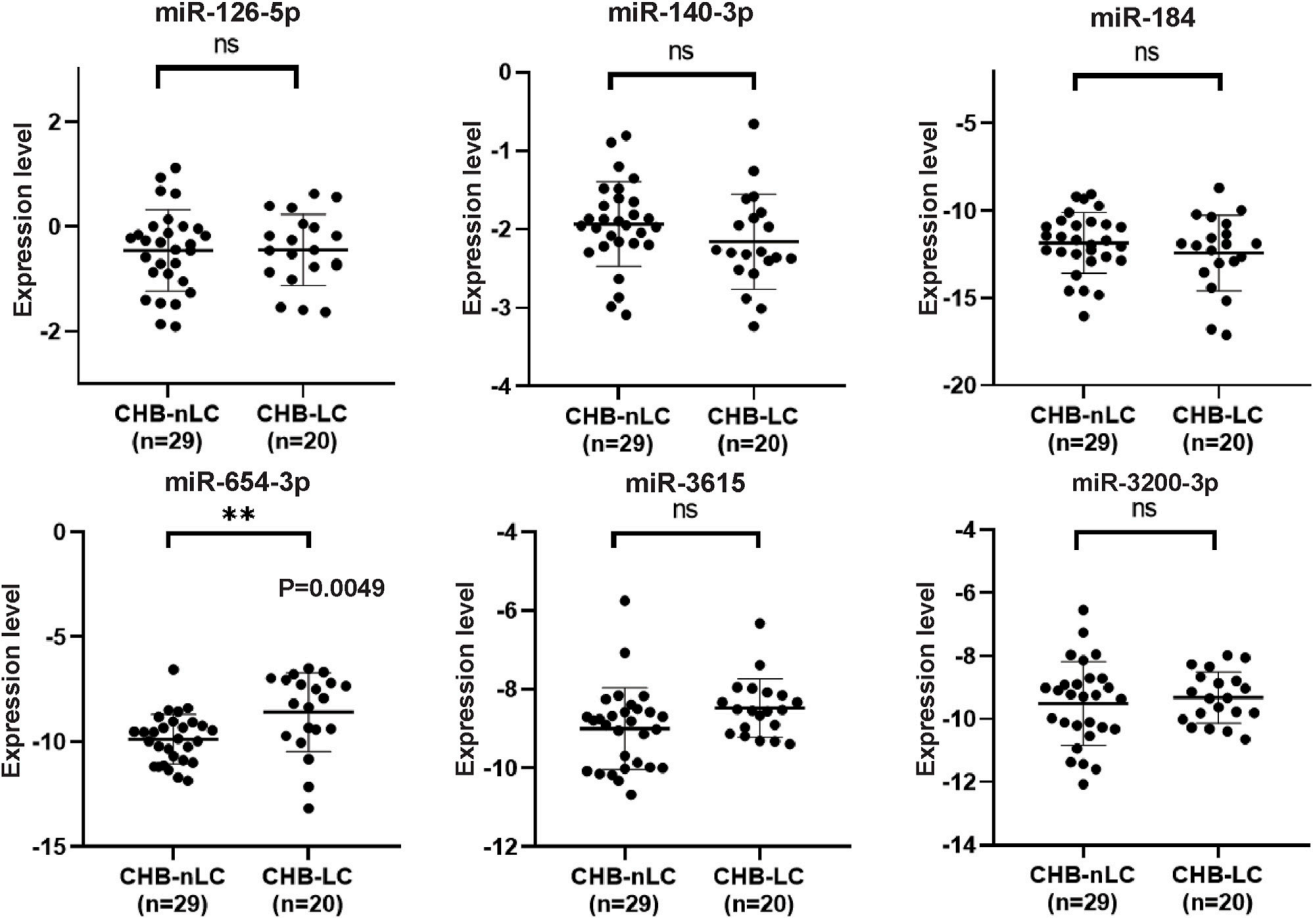
qRT-PCR analysis of six selected miRNAs in CHB-LC patients compared to CHB-nLC patients. The results demonstrate that miR-654-3p showed a significant upregulation in the plasma sEVs of CHB patients with liver cirrhosis. On the other hand, the expression levels of the other five candidate miRNAs remained unchanged.

The selection criteria for miRNAs were based on functional and pathway enrichment analysis, which indicated their involvement in tumor regulation. Additionally, published reports linking these miRNAs to cancer or hepatic diseases were considered. Any miRNA that met these criteria was included in the selection process. Considering the challenges in extracting low-abundance miRNAs from peripheral blood, which are sensitive to extraction techniques and experimental conditions, miRNAs with counts less than 100 were excluded from subsequent validation procedures (Supplementary Figure S1). As a result, the candidate miRNAs that fulfilled the criteria and were chosen for further analysis were miR-126-5p, miR-140-3p, miR-184, miR-654-3p, miR-3615, and miR-3200-3p. To confirm the specific expression of these miRNAs in HCC patients, we collected additional samples, including 77 HCC samples and 100 non-HCC (nHCC) samples (comprising CHB patients and healthy donors). qRT‒PCR revealed that miR-140-3p (*p* = 0.0001) and miR-3200-3p (*p* = 0.0017) were lower in the HCC samples (Figure 3), indicating their potential as HCC-specific miRNAs. Furthermore, we validated the expression of these candidate miRNAs in CHB patients. Compared to those in CHB patients without liver cirrhosis (CHB-nLC), sEV-delivered miR-654-3p levels were upregulated in CHB-LC patients (*p* = 0.0049; Figure 4).

### 3.4 Prediction of target genes of verified miRNAs and functional enrichment analysis

**FIGURE 5:**
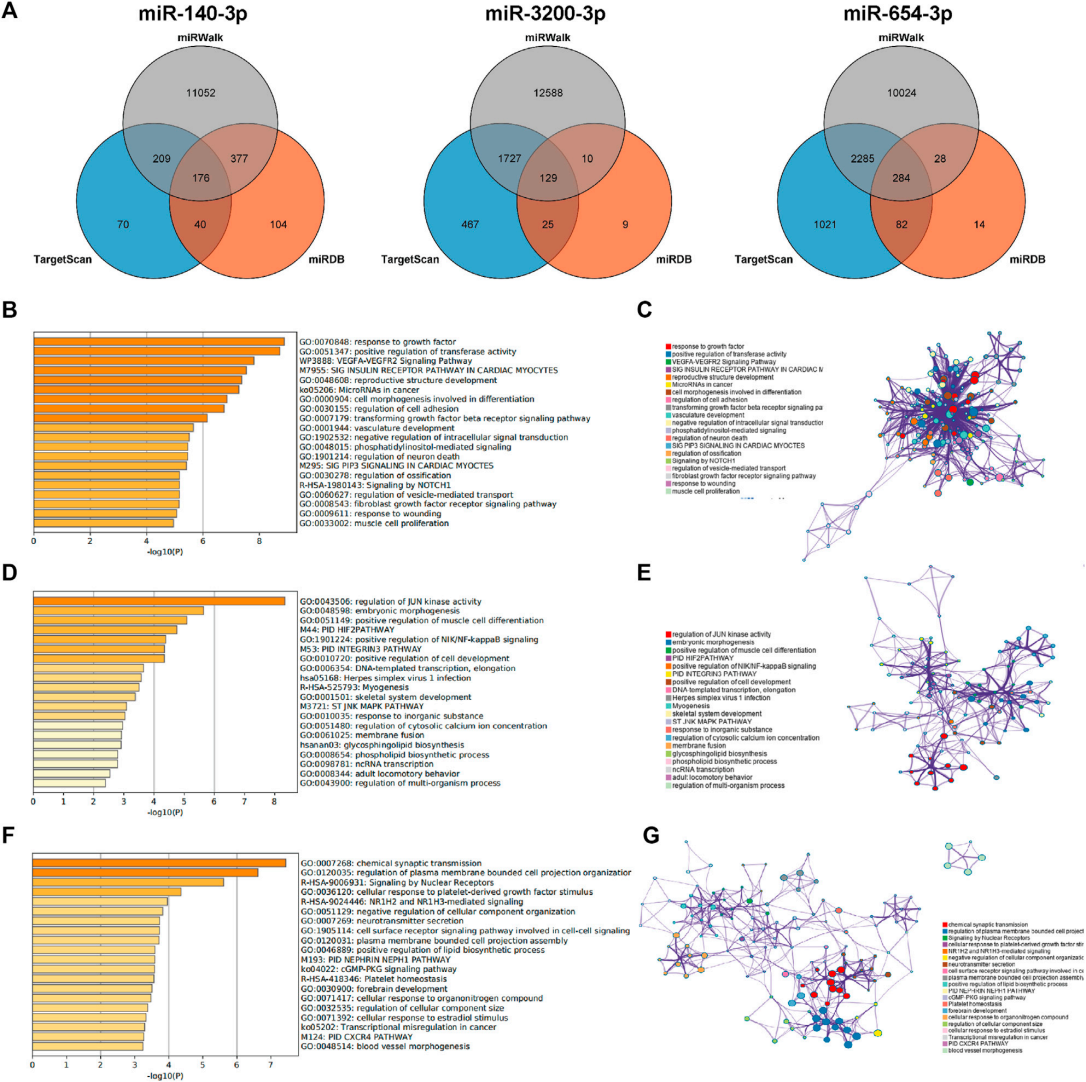
Prediction of target genes. **(A)** Venn diagram of the common predicted genes for the three miRNAs. **(B,D, F)** GO enrichment analysis for miR-140-3p, miR-3200-3p, and miR-654-3p, respectively. The top 20 clusters are shown in the bar graph based on the list of input genes, with the color representing the *p*-values. The log10(P) indicates the *p*-value in logarithmic base 10. **(C,E,G)** Network visualization of the selected enriched terms. The top 20 informative entries from the obtained GO clusters are depicted. Nodes in the network are colored based on their cluster IDs.

To elucidate the functions and roles of the three validated DEMs, multiple algorithms were used. The genes predicted by all three algorithms were selected as the final target genes for each miRNA. Specifically, 176 target genes were predicted for miR-140-3p, 129 for miR-3200-3p, and 284 for miR-654-3p based on the combined analysis of the three software programs (Figure 5A).

To further explore the functions associated with these target genes, pathway and process enrichment analyses were conducted using the Metascape database. The results revealed significant associations for the target genes of each miRNA. The target genes of miR-140-3p were involved in processes such as the response to growth factors, positive regulation of transferase activity, the VEGFA-VEGFR2 signaling pathway, microRNAs in cancer, and the transforming growth factor beta (TGF-beta) signaling pathway (Figures 5B,C). The target genes of miR-3200-3p were associated with JUN kinase activity, embryonic morphogenesis, primary immunodeficiency (PID), positive regulation of NIK/NF-kappaB signaling, and DNA-templated transcription and elongation (Figures 5D,E). The target genes of miR-654-3p were primarily involved in plasma membrane and cell projection organization; signaling by nuclear receptors; NR1H2- and NR1H3-mediated signaling; platelet-derived growth factor; and positive regulation of lipid biosynthetic processes (Figures 5F,G). These findings indicate a strong correlation between the identified target miRNAs and the progression of liver cancer.

### 3.5 HCC-related risk factors

Conditional logistic regression analysis was used to evaluate the correlation between the selected DEMs and the risk of HCC. The results, presented in Table 2, indicated that there was a significantly increased risk for HCC associated with the expression of miR-140-3p (OR = 0.377, 0.217–0.655) and miR-3200-3p (OR = 0.622, 0.448–0.861). Other miRNAs did not significantly change in relation to the risk of HCC. These findings further support the idea that miR-140-3p and miR-3200-3p may have substantial implications for the risk of developing HCC.

**TABLE 2:** Differentially expressed miRNAs associated with risk of HCC.

miRNAs	Case number	*p*-value	OR	95%CI
miR-126-5p	177	0.098	1.425	0.937–2.166
miR-140-3p	177	0.001	0.377	0.217–0.655
miR-184	177	0.948	1.005	0.870–1.161
miR-654-3p	177	0.331	0.907	0.744–1.105
miR-3615	177	0.090	1.328	0.957–1.843
miR-3200-3p	177	0.004	0.622	0.448–0.861

Abbreviations: OR, odd ratio; CI, confidence interval.

Furthermore, we performed correlation analysis among clinical and laboratory indicators within the same patient cohort. The findings revealed that HBsAg, AFP, CEA, ALT, AST, ALP, GGT, and MONO% were positively associated with HCC risk. Conversely, HBsAb, HBeAb, HBcAb, TP, ALB, RBC, HB, HCT, and LYMPH% were negatively correlated with HCC risk (Table 3).

**TABLE 3:** Clinical and laboratory indicators associated with risk of HCC.

Indicators	*p*-value	OR	95%CI	Indicators	*p*-value	OR	95%CI
HBsAg	0.000	1.348	1.231–1.424	UA	0.048	0.993	0.987–1.000
HBsAb	0.014	0.997	0.994–0.999	TG	0.023	0.296	0.103–0.847
HBeAg	0.699	0.997	0.984–1.011	TC	0.057	0.634	0.396–1.014
HBeAb	0.030	0.485	0.252–0.934	HDL	0.010	0.096	0.016–0.575
HBcAb	0.012	0.353	0.157–0.797	LDL	0.227	0.733	0.443–1.213
AFP	0.001	1.406	1.303–1.610	CK	0.051	0.990	0.981–1.000
CEA	0.015	1.554	1.088–2.219	LDH	0.335	0.997	0.992–1.003
PIVKAⅡ	0.065	1.001	1.000–1.003	HBDH	0.263	0.995	0.986–1.004
TBIL	0.638	0.990	0.950–1.032	RBC	0.005	0.412	0.221–0.770
DBIL	0.676	0.997	0.981–1.013	HB	0.001	0.963	0.942–0.984
IBIL	0.111	0.947	0.885–1.013	HCT	0.001	0.000	0.000–0.004
ALT	0.027	1.009	1.001–1.016	MCV	0.360	0.977	0.929–1.027
AST	0.014	1.011	1.002–1.019	MCH	0.148	0.912	0.806–1.033
ALP	0.002	1.012	1.004–1.020	MCHC	0.062	0.974	0.947–1.001
GGT	0.000	1.028	1.014–1.043	PLT	0.030	0.995	0.990–0.999
TP	0.000	0.884	0.828–0.945	WBC	0.215	0.911	0.787–1.055
ALB	0.000	0.779	0.707–0.858	NEUT%	0.151	1.023	0.922–1.055
BUN	0.396	0.857	0.600–1.224	LYMPH%	0.038	0.961	0.926–0.998
CREA	0.075	0.965	0.928–1.004	MONO%	0.000	1.417	1.169–1.718
Cys C	0.154	3.658	0.614–21.784	EO%	0.320	0.400	0.832–1.062

Abbreviations: HBsAg, hepatitis B surface antigen; HBsAb, hepatitis B surface antibody; HBeAg, hepatitis B e antigen; HBeAb, hepatitis B e antibody; HBcAb, hepatitis B core antibody; AFP, alpha fetoprotein; CEA, carcinoembryonic antigen; PIVKAⅡ, prothrombin induced by vitamin K absence or antagonist-II; TBIL, total bilirubin; DBIL, direct bilirubin; IBIL, indirect bilirubin; ALT, alanine transaminase; AST, aspartate aminotransferase; ALP, alkaline phosphatase; GGT, gamma-glutamyl transpeptidase; TP, total protein; ALB, albumin; BUN, blood urea nitrogen; CREA, creatinine; Cys-C, cystatin C; UA, ursolic acid; TG, triglyceride; TC, total cholesterol; HDL, high-density lipoprotein; LDL, low-density lipoprotein; LDH, lactate dehydrogenase; HBDH, hydroxybutyrate dehydrogenase; RBC, red blood cell count; HB, hemoglobin; HCT, hematocrit; MCV, mean red blood cell volume; MCH, mean erythrocyte hemoglobin; MCHC, mean erythrocyte hemoglobin; PLT, platelet count; WBC, white blood cell count; NEUT%, neutrophil percentage; LYMPH%, lymphocyte percentage; MONO%, monocyte percentage; EO%, eosinophil percentage.

### 3.6 The diagnostic value of the selected variables for HCC

**FIGURE 6:**
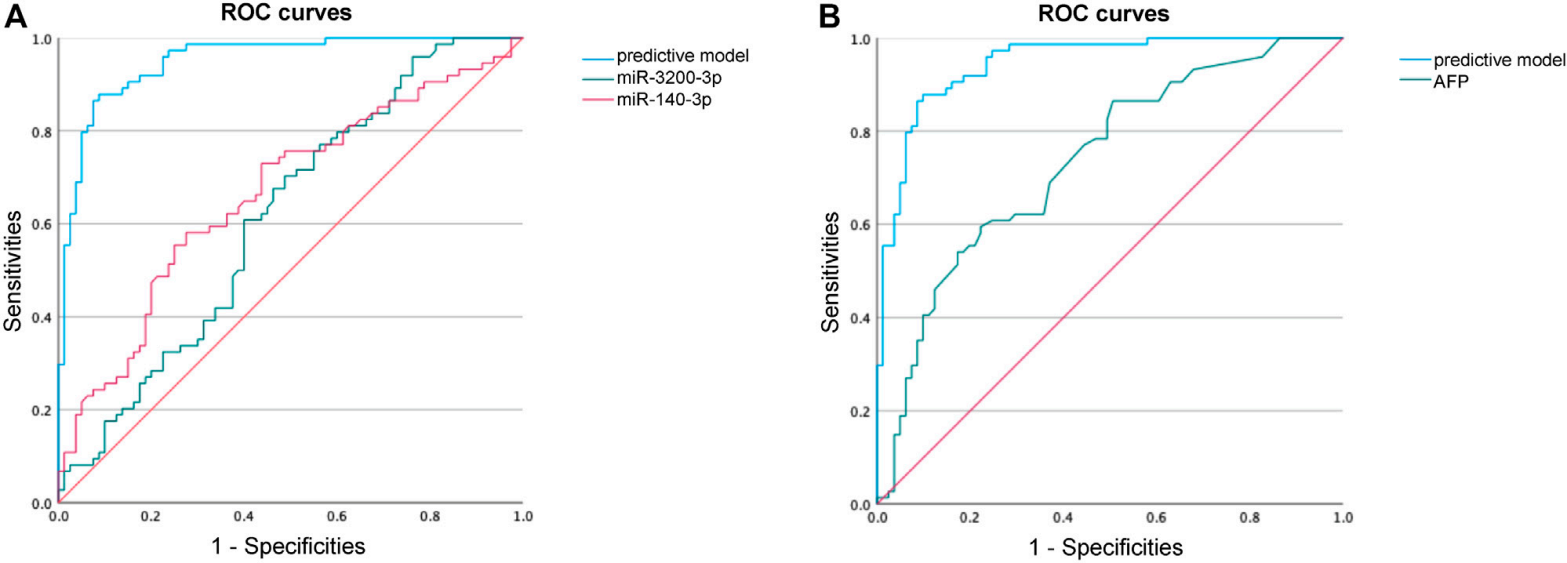
ROC curve analysis to identify HCC. **(A)** ROC curve performance of our predictive model compared with miR-140-3p and miR-3200-3p. **(B)** ROC curve performance of our predictive model compared with AFP.

To evaluate the diagnostic potential of the predictive model, we generated ROC curves and calculated AUCs. The enhanced predictive model included AFP>20, miR-140-3p, miR-3200-3p, HBsAg, and age as the independent variables. At the optimal cutoff value, the predictive model demonstrated exceptional performance, achieving 90.1% specificity and 87.8% sensitivity, along with an AUC of 0.951 (Figure 6A; Table 4).

**TABLE 4:** The diagnostic values of different models.

	AUC	95%CI	Cut-off	Sensitivity	Specificity	Youden index	+LR	-LR
Predictive model	0.951	0.919–0.983	0.493	0.878	0.901	0.779	8.869	0.135
miR-3200-3p	0.614	0.525–0.720	0.108	0.703	0.512	0.215	1.441	0.580
miR-140-3p	0.665	0.579–0.751	0.431	0.581	0.725	0.306	2.113	0.578
AFP	0.737	0.658–0.815	31.500	0.595	0.778	0.373	2.680	0.521

Abbreviations: AUV, area under curve; +LR, positive likelihood ratio; -LR, negative likelihood ratio.

Our predictive model significantly outperformed the diagnostic accuracy of using miR-140-3p alone (AUC = 0.665, 95% CI 0.579–0.751) or miR-3200-3p alone (AUC = 0.614, 95% CI 0.525–0.720) for distinguishing HCC between HCC patients and healthy controls (Figure 6A; Table 4). Additionally, we evaluated the diagnostic performance of AFP for distinguishing HCC patients from non-HCC patients and found that AFP alone has limited ability (AUC = 0.737, 95% CI 0.658–0.815). These results suggest that combining AFP with sEV-delivered miRNAs may provide a valuable diagnostic model for HCC.

## 4 Discussion

Communication between cells via EVs is crucial in normal physiological processes as well as disease conditions since the quantity and composition of EVs can reflect the aberrant state of donor cells (Sung et al., 2018). The cargo carried by EVs plays pivotal roles in regulating the tumor microenvironment (TME) and holds promise as a potential disease biomarker.

In this study, we investigated differentially expressed miRNAs between the HCC plasma-derived sEV and that from the nHCC patients. After validation via qRT‒PCR, miR-140-3p and miR-3200-3p emerged as distinguishing HCC miRNAs. GO analysis confirmed their enrichment in cancer-related pathways, indicating their potential roles in cancer occurrence and progression. Finally, by integrating clinicopathological data, we constructed a diagnostic model. Our most significant finding in the present study was that our model exhibited a significantly improved ROC curve for diagnosing HCC compared to serum AFP levels. Our model achieved an AUC of 0.951, with 90.1% specificity and 87.8% sensitivity at the optimal cutoff, confirming its high diagnostic accuracy. This substantial enhancement overcomes the limitations of AFP in diagnosing HCC.

In our study, we observed lower levels of miR-140-3p in sEVs from HCC patients than in those from. miR-140-3p is recognized as a tumor suppressor gene and is known to exhibit decreased expression in various types of tumors. Previous reports have indicated that decreased expression of miR-140-3p in HCC is associated with epithelial-mesenchymal transition (EMT) and tumor cell invasion and migration (Wen et al., 2020; Gao et al., 2021). However, relatively few studies have investigated on the expression levels and functions of sEV-delivered miR-140-3p. Yu et al. reported that miR-140-3p was downregulated in sEVs derived from highly metastatic HCC cells compared to sEVs derived from less metastatic HCC cells (Yu et al., 2019). Tumor invasion and metastasis are closely associated with poor prognosis. Yu et al. further analyzed the correlation between overall survival (OS) and miR-140-3p levels and revealed improved OS in patients with high miR-140-3p expression. These findings suggest that miR-140-3p in sEVs may serve as a candidate biomarker for predicting HCC cell migration and prognosis, potentially guiding the treatment of advanced-stage HCC. In addition to its effect on HCC, miR-140-3p has been found to significantly decrease in the serum sEVs of patients with multiple myeloma (Zhang et al., 2019). In nasopharyngeal carcinoma (NPC) patients, decreased levels of miR-140-3p in serum contribute to the early diagnosis of NPC, although there is no difference in the expression of serum-derived sEVs (Zhang et al., 2020; Zou et al., 2020). It is important to note that 90% of circulating miRNAs are not transported in EVs but are rather stably bound to the Argonaute2 (Ago2) complex (Arroyo et al., 2011). This difference may explain the differences in miRNA expression levels between serum and EVs.

The nuclear factor-κB (NF-κB) transcription factor family mediates the inflammatory process and is implicated in cancer initiation and progression by regulating miRNA expression (Karin, 2006). miR-3200-3p was found to be a target of NF-κB, suggesting its significant role in the NF-κB pathway (Zhou et al., 2014). Huang et al. associated miR-3200-3p with recurrence after liver transplantation (RALT) and poor overall survival (OS) (Huang et al., 2021). miR-3200-3p levels are downregulated in glioblastoma cells (Wang et al., 2022). This downregulation of miR-3200-3p reduces CAMK2A expression, enhances cell proliferation and colony formation, and significantly promotes glioblastoma cell migration. These findings indicate that miR-3200-3p not only regulates cell proliferation and migration but is also correlated with tumor recurrence. In recent years, several studies have reported the presence of miR-3200-3p in sEVs, indicating its involvement in tumor progression. Hui et al. discovered that inhibiting VEGFR2 promotes the expression of miR-3200-3p in tumor cell-secreted sEVs, leading to regulatory T-cell (Treg) aging in the TME and inhibiting subcutaneous tumor growth (Hui et al., 2024). Cho et al. identified a correlation between the downregulation of miR-3200-3p and the early progression of cervical cancer during radiotherapy (Cho et al., 2021). The decreased expression of miR-3200-3p in the plasma sEVs of HCC patients further underscores the importance and clinical potential of this miRNA.

Despite promising results, our candidate miRNAs have limitations as diagnostic biomarkers in clinical practice. The molecular mechanisms of distinct histologic subtypes of HCC may differ, leading to variations in circulating sEV levels. Additionally, the small sample size in this study necessitates further investigation with larger sample sizes to validate the current findings. Considering that patients with HCC, cirrhosis, and HBV receive diverse therapies, it is important to determine whether different therapy modalities affect the optimal specificity and sensitivity of validated miRNAs. To fully understand the mechanisms of miRNAs in liver diseases, additional experimental studies are needed. Moreover, rigorous inclusion criteria for patients in different groups are essential to bolster these conclusions. Thus, these aspects warrant exploration in future studies.

## 5 Conclusion

In summary, our study effectively isolated sEVs from samples and characterized the miRNA expression profile of HCC plasma-derived sEVs. Our findings indicate that miR-140-3p and miR-3200-3p expression is lower in HCC patients. The constructed model demonstrated promising diagnostic potential, as revealed by ROC curve analysis. However, further comprehensive studies are required to assess their actual performance.

## Data Availability

The data that support the findings of this study have been deposited into CNGB Sequence Archive (CNSA) of China National GeneBank DataBase (CNGBdb) with accession number CNP0005793.

## Supplementary material

SUPPLEMENTARY FIGURE S1 The expression levels of the 9 feature miRNAs identified by random forest algorithm. The x-axis represents different groups, and the y-axis represents the counts of each miRNA.
