# Supplementary figures and images for: Diagnostic model for hepatocellular carcinoma using small extracellular vesicle-propagated miRNA signatures
Source: Front Mol Biosci. 2024 Jun 28;11:1419093. doi: 10.3389/fmolb.2024.1419093 (PMC11239443; doi:10.3389/fmolb.2024.1419093)

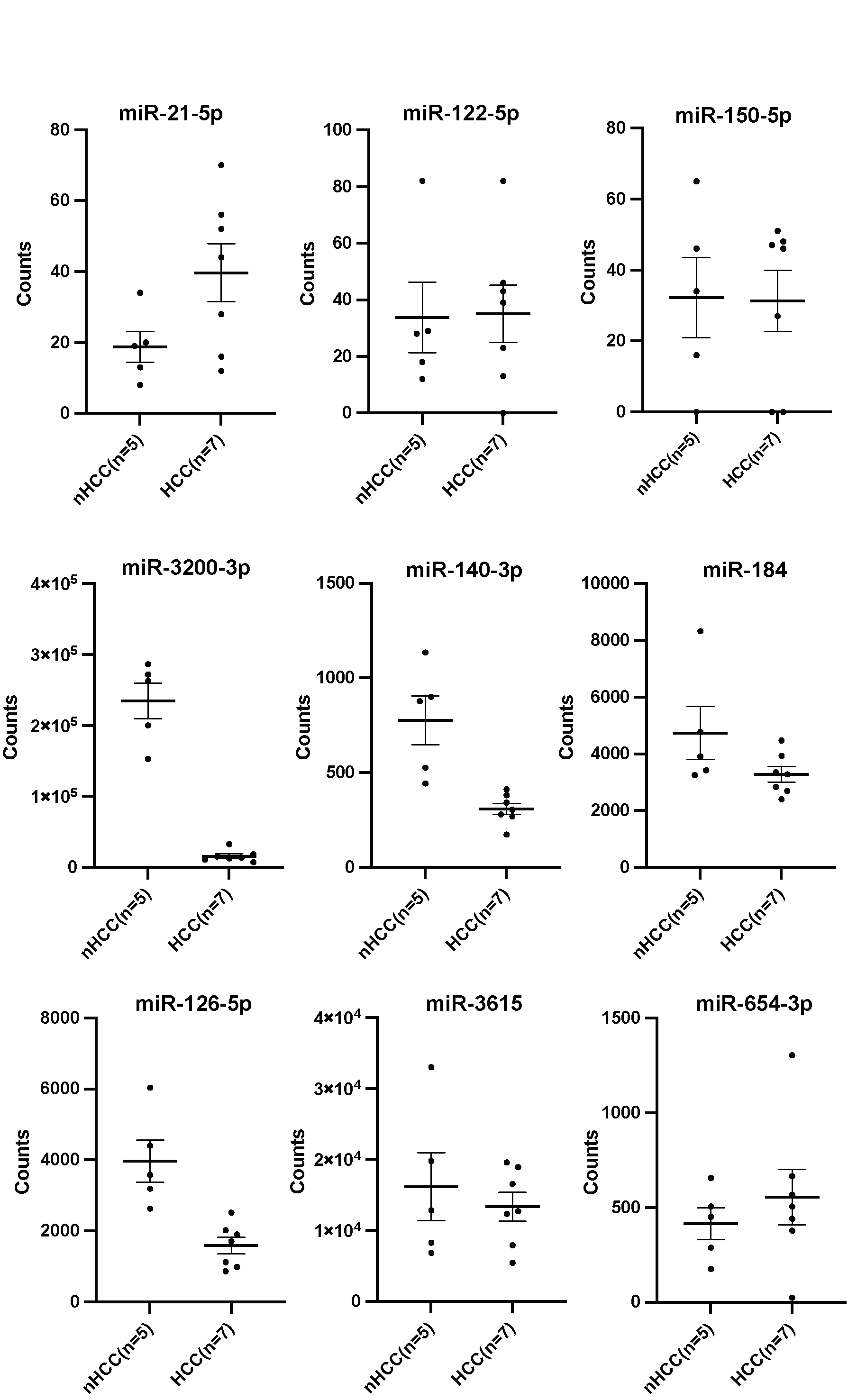

Supplement: Supplementary file 3 [file Image1.TIF]
